# Maui Wildfire and 988 Suicide and Crisis Lifeline Call Volume and Capacity

**DOI:** 10.1001/jamanetworkopen.2024.46523

**Published:** 2024-11-20

**Authors:** Alexandra C. Rivera-González, Jonathan Purtle, Joseph Keawe‘aimoku Kaholokula, Jim P. Stimpson, Alexander N. Ortega

**Affiliations:** 1 Department of Public Health, School of Social Sciences, Humanities, and Arts, University of California, Merced; 2 Department of Public Health Policy and Management, School of Global Public Health, New York University, New York; 3 Department of Native Hawaiian Health, John A. Burns School of Medicine, University of Hawai‘i at Mānoa, Honolulu; 4 Peter O’Donnell Jr. School of Public Health, University of Texas Southwestern Medical Center, Dallas; 5 Thompson School of Social Work & Public Health, University of Hawai‘i at Mānoa, Honolulu, Hawaii

## Abstract

This cross-sectional study examines 988 Suicide Crisis Lifeline Call data after the Maui wildfire in August 2023.

## Introduction

In August 2023, the island of Maui in Hawai‘i had a wildfire that destroyed the historic town of Lāhainā, displaced tens of thousands of people, and resulted in at least 101 deaths.^1^ Since the wildfire, Maui remains in a mental health crisis, with an increase in depressive, anxiety, and substance use disorders, retraumatization by tourists and government officials, and homelessness, all of which have heightened concerns over suicide risk. Nationally launched in July 2022, the 988 Suicide and Crisis Lifeline (988 Lifeline) has the potential to mitigate psychological effects of disasters and provide timely estimates of mental health services demand,^2^ especially for people with serious or moderate psychological distress.^3^ Comparing Hawai‘i with other states and territories, this study analyzed 988 Lifeline call data before and after the wildfire to observe its association with call volume, reflecting demand for mental health services, and in-state answer rate, a measure of quality and service capacity.^4^

## Methods

Data from 988 Suicide and Crisis Lifeline calls from July 2022 to August 2024 were compiled from publicly available state-based and networkwide monthly reports by Vibrant Emotional Health.^2^ The study was deemed exempt from institutional review board review by the University of Hawai‘i Office of Research Compliance, and informed consent was not required because observations were deidentified. This cross-sectional study followed Strengthening the Reporting of Observational Studies in Epidemiology (STROBE) reporting guideline. The primary outcome was call rate per 100 000 population, which was estimated using the number of calls routed to a 988 Lifeline call center in Hawai‘i based on caller area code. If the wait time at a local center is too long because of insufficient staffing capacity to meet call volume demand, the call is routed to a national overflow 988 Lifeline call center; thus, the secondary outcome was the percentage of calls answered in-state.

Monthly call volume and in-state answer rate trends were plotted for the study period, incorporating a postwildfire indicator (August 2023 onwards). Networkwide estimates reflecting all other states and territories, excluding Hawai‘i, were used as comparisons. Using Stata’s eventdd command, event study analyses were performed for call and answer rates separately using a fixed-effects regression to account for seasonality and unobserved heterogeneity of groups (Hawai‘i vs networkwide excluding Hawai‘i). Significance was measured using α = .05, and tests were 2-sided. Analyses were performed using StataNow/MP version 18.5 (StataCorp). Data were analyzed from July to September 2024.

## Results

**Figure 1.**
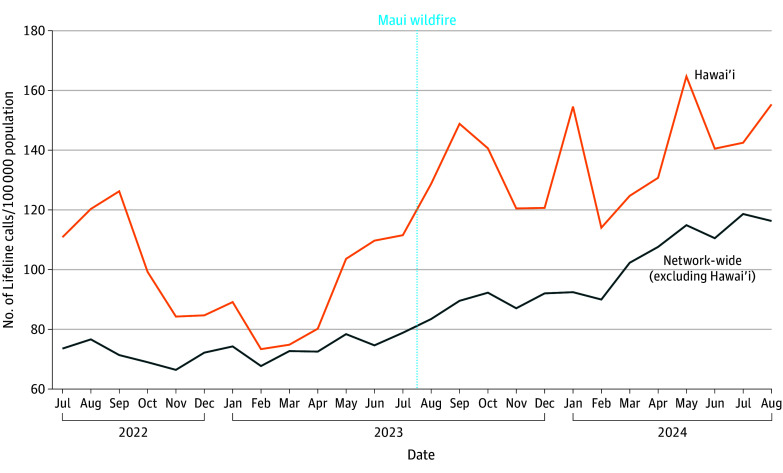
Volume Rates for 988 Calls: Hawai‘i vs Other States and Territories

During the study, total monthly 988 Suicide and Crisis Lifeline calls routed to a Hawai‘i call center ranged from 1018 to 2277. The 988 Suicide and Crisis Lifeline call rate increased from a monthly mean (SD) of 97.5 (17.6) calls per 100 000 Hawai‘i residents during the prewildfire period to 137.4 (15.6) calls per 100 000 residents in the postwildfire period (Figure 1). After holding other factors constant, the call rate increased by a mean (SE) of 13.1 (5.9) calls per 100 000 residents in Hawai‘i following the wildfire (*P* = .04). Positive and substantial lag times were detected for months 9 to 12 postwildfire, which suggests a sustained increase in call volume following the disaster.

**Figure 2.**
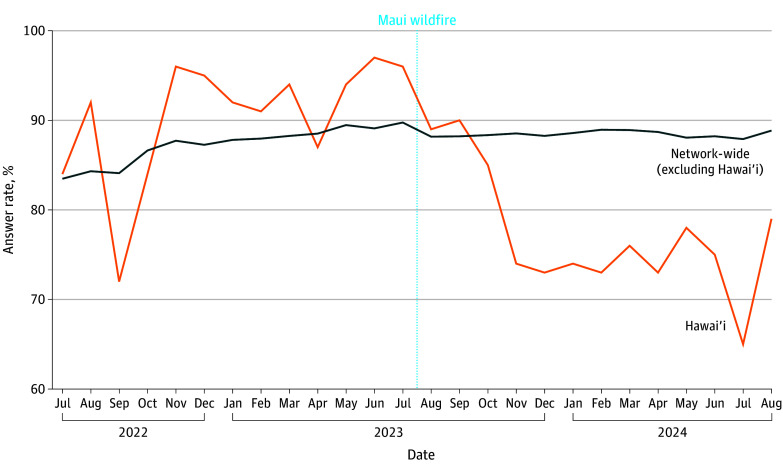
In-State Answer Rates for 988 calls: Hawai‘i vs Other States and Territories

In-state answer rates for Hawai‘i ranged from 1276 of 1969 calls (65%) to 1477 of 1520 calls (97%) during the study period. The in-state answer rate for Hawai‘i decreased from a monthly mean (SD) of 90.3% (7.0%) during the prewildfire period to 77.2% (7.0%) in the postwildfire period (Figure 2). The answer rate significantly decreased by a mean (SD) of 14.3% (2.5%) (*P* < .001) in Hawai‘i following the wildfire, after holding other factors constant.

## Discussion

Maui has had a growing mental health crisis since the August 2023 wildfire.^5^ The increase in calls to the 988 Lifeline highlight the high demand for mental health services after the wildfire. Mental health professionals from neighboring islands and outside of Hawai‘i have helped to meet this demand, but on-island mental health care capacity is critical for the long-term mental health needs of residents.^6^

A limitation of this study is that calls to 988 Lifeline from Hawai‘i residents with a non-808 area code are not counted toward Hawai‘i’s state-level rates and are not specific to Maui. Nonetheless, the results indicate the need for a focus on postwildfire mental health services inequities and greater mental health service capacity in Maui following the disaster.

## Notes

Supplement.
Data Sharing Statement
