# Supplementary material for: Maui Wildfire and 988 Suicide and Crisis Lifeline Call Volume and Capacity
Source: JAMA Netw Open. 2024 Nov 20;7(11):e2446523. doi: 10.1001/jamanetworkopen.2024.46523 (PMC11579797; doi:10.1001/jamanetworkopen.2024.46523)
Supplement: Supplement. — Data Sharing Statement [file jamanetwopen-e2446523-s001.pdf]

Data Sharing Statement

Data
Data available: No

Additional Information
Explanation for why data not available: Data is publicly available via Vibrant Health (https://988lifeline.org/our-network/).
